# Efficient Characterization and Classification of Contrast Sensitivity Functions in Aging

**DOI:** 10.1038/s41598-017-05294-0

**Published:** 2017-07-11

**Authors:** Fang-Fang Yan, Fang Hou, Zhong-Lin Lu, Xiaopeng Hu, Chang-Bing Huang

**Affiliations:** 1 CAS Key Laboratory of Behavioral Science, Institute of Psychology, Chinese Academy of Sciences, Beijing, 100101 China; 2 Department of Psychology, University of Chinese Academy of Sciences, Beijing, 100101 China; 3 Laboratory of Brain Processes (LOBES), Center for Cognitive and Brain Sciences, Center for Behavioral and Cognitive Brain Imaging, and Department of Psychology, The Ohio State University, Ohio, 43210 USA; 4 Department of Radiology, The First Affiliated Hospital of Anhui Medicial University, HeFei, Anhui 230022 China

## Abstract

The contrast sensitivity function (CSF), delineating contrast sensitivity over a wide range of spatial frequencies, provides a comprehensive characterization of spatial vision and a sensitive test for many physiological and pathological processes. A precise CSF measurement tool for the aging population is of great theoretical and practical importance. In the current study, we tested whether the assumptions of the newly developed quick CSF method were valid and whether it can rapidly, reliably, and effectively evaluate CSFs in the aging population. The quick CSF method combines Bayesian adaptive inference with an information gain strategy to directly estimate four parameters that define the observer’s CSF. Eighteen young and twenty-five old observers participated in the evaluation of the quick CSF method. All observers were screened for ophthalmological and mental diseases. Our results showed that the CSFs derived from the quick CSF method well matched with those from the traditional Ψ method, with excellent test-retest reliability. More importantly, the quick CSF method can accurately detect the difference of CSFs between the young and old groups. Aging significantly degrades contrast sensitivity. The quick CSF method demonstrates great potentials for both laboratory research and clinical applications in the aging population.

## Introduction

Aging is an irreversible stage of human life. Aging, either normal or pathological, impairs a variety of visual functions, including visual acuity^1, 2^, contrast sensitivity^3–5^, motion perception^6–9^, orientation discrimination^10^, and contour integration^11, 12^. Age-related vision decline or impairment affects the quality of life^13, 14^. It increases the probability of falls in locomotion^15, 16^ and crash risk during driving^17^, and has been related to age-related declines in other cognitive and behavioral functions. For example, Lin and colleagues^18^ found that impairment of distant visual acuity was associated with cognitive retardation in a large sample of older women. Salthouse, Hancock, Meinz and Hambrick^19^ found that impairment of near visual acuity significantly affected working memory, associative learning and concept identification.

Among all the affected visual functions, contrast sensitivity, or sensitivity to luminance variations, is one of the most fundamental functions of the visual system^20^. Delahunt, Hardy, and Werner^21^ found that the decline of contrast sensitivity in the elderly could account for increased orientation discrimination thresholds. Greene and Madden^22^ showed that only contrast sensitivity remained significantly different between old and young observers after taking into account the correlations among near acuity, distant acuity, stereopsis, and contrast sensitivity. In addition, many studies showed that even after visual acuity was compensated, contrast sensitivity was still impaired by aging^3–5^. These studies highlight the importance and necessity of evaluating contrast sensitivity in monitoring age-related vision decline.

The contrast sensitivity function (CSF), delineating contrast sensitivity over a wide range of spatial frequencies, provides a comprehensive characterization of spatial vision and a sensitive test for many physiological and pathological processes^23^. Clinical CSF tests, e.g. the Pelli-Robson chart^24^, Vistech contrast sensitivity chart^25^ and its descendants, the Functional Acuity Contrast Test (FACT)^26^, the Contrast Sensitivity Tester 1800 (Vision Sciences Research Corporation, San Ramon, CA), and CVS-1000 series chart (VectorVision Dayton, OH) can be used to get a rough estimate of the CSF but are not very precise because of the coarse sampling of stimulus contrast and spatial frequency^27, 28^; laboratory implementations of CSF measurements using more objective psychophysical methods are usually time-consuming (i.e., requiring hundreds of trials) and impractical for clinical applications in special populations. Lesmes, Lu, Baek, and Albright^29^ developed the quick CSF method based on the Bayesian adaptive framework to measure CSF with as few as 50 trials by optimally sampling both stimulus contrast and spatial frequency to maximize information gain over the CSF parameters in each trial. The quick CSF method has been successfully applied to measure CSF in both foveal^29–31^ and peripheral vision^32^ of normal observers, and in foveal vision of amblyopic observers^33^.

The quick CSF method has two important assumptions^29^. First it assumes that a CSF curve can be described by a four-parameter truncated log-parabola^34^. The second assumption is that the slope of the psychometric functions^35–37^ across all the spatial frequencies and observers is fixed. If they were invalid, these assumptions would adversely affect the performance of the method. Model mismatch between the assumed properties of the CSF in the quick CSF method and the actual properties of the observer can introduce systematic bias into the estimated CSFs.

It has been reported that the contrast sensitivity in aging exhibited more prominent loss in high spatial frequencies than in low spatial frequencies^38–40^. This could lead people to question the validity of the truncated log parabola model of the CSF in the aging population. Furthermore, Bennett, Sekuler, Ozin^41^ reported that the calculation efficiency was lower for old than for young observers. Pardhan^42^ recently found that, compared with young subjects, older subjects showed lower sampling efficiencies at low spatial frequencies and higher equivalent noise levels in high spatial frequencies, indicating that the neural and optical factors might affect contrast sensitivity loss with aging differently. These results suggest that we must carefully examine the fixed slope assumption of the quick CSF method. Taken together, a rigorous test and accompanying analysis have to be carried out before we can apply the quick CSF method in the aging population.

In this study, we tested whether the quick CSF method can accurately and efficiently assess the CSF in the aging population by comparing CSFs obtained with the quick CSF method with those obtained with the traditional Ψ method^43^, and evaluated the test-retest reliability of the quick CSF method through repeated measurements at both intermediate and far viewing distances. The results showed that the quick CSF method with 50~100 trials can effectively characterize the contrast sensitivity function in the aging population and discriminate the aged from the young control group at both distances. The underlying assumptions of the quick CSF method were valid in the aging population.

## Methods

The study consisted of two experiments, one performed at a viewing distance of 1.86 meters (Experiment 1, intermediate distance) and the other at 4.29 meters (Experiment 2, far distance), to evaluate the effects of presbyopic blur^44, 45^ on the validity of quick CSF methods. All experimental setups and subject inclusion criteria, unless otherwise noted, were the same in the two experiments.

### Observers

Ten young observers (22.6 ± 2.7 yrs, mean ± sd.) and seventeen old observers (68.4 ± 4.9 yrs) participated in Experiment 1. Eight young observers (24.0 ± 2.1 yrs) and eight old observers (65.5 ± 4.8 yrs) participated in Experiment 2 (see Table 1 for details). No significant difference in visual acuity and age was found between subjects in the two respective age groups in Experiments 1 and 2 (both p > 0.10). The young observers were recruited from universities, and the old observers were from nearby communities. All observers had normal or corrected-to-normal distant vision and were naive to psychophysical experiments. One ophthalmologist examined all the subjects. The inclusion criteria for participating in the experiment were: (1) visual acuity better than 20/25; (2) normal ocular media and free of retinal disease (e.g., strabismus, glaucoma, cataract, and macular degeneration); (3) normal trichromatic vision. The old observers were also free from problems associated with diabetes, experience of burning of strong beams on the eyes, history of mental diseases, and cognitive deficits (Mini-Mental State Examination (MMSE) > 24 points), and had a mean education level of 13.2 ± 3.2 years. Written informed consent was obtained from each observer after the nature of the study was explained. The experimental protocol was approved by the ethics committee of Institute of Psychology, Chinese Academy of Sciences and all research activities adhered to the tenets of the Declaration of Helsinki.

**Table 1:** Observer characteristics.

Experiment 1								Experiment 2							
Old Group				Young Group				Old Group				Young Group			
Obser	Sex	Age	Acuity	Obser	Sex	Age	Acuity	Obser	Sex	Age	Acuity	Obser	Sex	Age	Acuity
O1	F	76	−0.10	Y1	F	20	−0.11	O18	F	64	−0.01	Y11	F	23	−0.21
O2	M	75	−0.12	Y2	F	19	−0.11	O19	M	60	−0.11	Y12	F	24	−0.21
O3	M	70	−0.04	Y3	M	24	−0.10	O20	M	69	−0.05	Y13	M	20	−0.03
O4	F	62	0.03	Y4	M	21	−0.31	O21	M	61	−0.04	Y14	M	23	−0.10
O5	F	71	−0.03	Y5	M	25	−0.23	O22	F	66	−0.14	Y15	F	27	−0.21
O6	F	64	−0.10	Y6	M	26	−0.23	O23	M	75	−0.11	Y16	M	25	−0.25
O7	F	63	−0.04	Y7	F	21	−0.11	O24	F	65	−0.05	Y17	F	25	−0.17
O8	M	72	−0.05	Y8	M	26	−0.23	O25	M	64	−0.21	Y18	F	25	−0.10
O9	M	73	0.03	Y9	M	20	−0.17								
O10	F	60	−0.03	Y10	M	24	−0.23								
O11	F	65	−0.11												
O12	F	69	−0.01												
O13	F	74	0												
O14	F	65	−0.05												
O15	F	71	−0.12												
O16	F	66	−0.03												
O17	M	66	−0.10												
Average			−0.05 ± 0.01			−0.18 ± 0.02			−0.09 ± 0.02			−0.16 ± 0.03			

Abbreviations: Obser = Observers; M = male; F = female; Distant Acuity: logMAR; Average: mean ± se.

### Apparatus and Stimuli

Experimental stimuli were generated using a computer running Matlab with PsychToolBox extensions^46, 47^ and presented on a DELL color monitor with a spatial resolution of 1600 × 1200 pixels and a refresh rate of 85 Hz. A special circuit was used to combine outputs from the red and blue channels of the graphics card to produce 14-bit gamma-corrected gray-level resolution^48^. The mean background luminance of the display was 30 cd/m^2^. A chin rest was used to minimize head movement during the experiment. Observers viewed the display binocularly in fovea in a dimly lighted room at a distance of 1.86 meters (Experiment 1) or 4.29 meters (Experiment 2). The viewing distance in Experiment 2 was comparable to the distance at which distant visual acuity was measured (5 meters).

The stimuli were oriented 3° × 3° sinewave gratings (45°clockwise, or 45°counter-clockwise). To minimize edge effects, a half-Gaussian ramp (σ = 0.5°) was used to blend the gratings into the background.

### Quick CSF Implementation

The quick CSF method assumes that the CSF curve can be characterized with a truncated log parabola with four parameters: (1) the peak gain (contrast sensitivity) *γ*_max_; (2) the peak spatial frequency *f*_max_; (3) the low-frequency truncation 𝛿; and (4) the bandwidth *β*–the function’s full-width at half maximum (in octaves). Different combinations of parameter values are assigned a prior as a four-dimensional probability density function (*pdf*). The *pdf* is updated using Bayes’ rule based on observer’s response in identifying the orientation of﻿﻿ a grating of a certain contrast and spatial frequency level in each trial. The contrast and spatial frequency of the stimulus in the next trial is chosen such that the expected entropy of the *pdf* after the trial would be the lowest among all possible stimuli (i.e., maximum information gain). In the current implementation, the contrast varied from 0.1% to 99% in steps of 1.5 dBs and spatial frequency was sampled from 0.5 to 16 c/d in steps of 3 dBs. For CSF parameters, the peak gain was sampled from 10 to 400 in steps of 0.1 dBs, the peak spatial frequency was sampled from 0.25 to 12 c/d in steps of 0.1 dBs, the low-frequency truncation was sampled from 0.2 to 2 in steps of 1.2 dBs and the bandwidth was sampled from 0.05 to 2 in steps of 1.2 dBs. The prior of the four parameters was generated using a 4-D Hyperbolic Secant distribution, with μ of log_10_(60), log_10_(2), log_10_(0.5), log_10_(0.5) and standard deviation of 2, 1, 1, 1 for the four parameters, respectively.

A log-Weibull psychometric function was adopted in threshold estimation:

1 $${P}_{c}=(1.0-\alpha )\,-\,(1.0\,-\,\alpha \,-\,\mu )\times \exp (-{10}^{\eta (lo{g}_{10}(c)-lo{g}_{10}({\rm{\tau }}))}),$$

where *P*_*c*_ is fraction correct, *α* is the lapse rate and set to be 0.02, *μ* is the guessing rate of 0.5, τ is the contrast threshold at 80.3% performance level, and *η* is the slope of the psychometric function and set to be 3.5^49^. Our results in the “Evaluating the Constant Slope Assumption” Section showed that exact matching of the assumed slope and the observer’s real slope is not critical for threshold estimation, consistent with Hou *et al*.^33^.

### Procedure

A grating orientation identification task was used. Each trial started with a 294-ms fixation cross in the center of the screen, with a brief tone signaling each trial’s onset. This was followed by a 153-ms blank screen, and a 141-ms grating stimulus that was tilted either 45° clockwise or 45° counter-clockwise from vertical with equal probability. Observers were asked to report the orientation of the grating by a key press. No feedback was provided. The next trial started after response and an inter-trial interval of 588 ms.

### Design

Observers were prescribed with 100 practice trials to get familiarized with the experimental setting and procedure. Each session of the main experiment had 600 trials, with 300 quick CSF trials and 300 Ψ method trials. For the Ψ method, we measured contrast sensitivity at six spatial frequencies (0.69, 1.29, 2.42, 4.54, 8.52 and 16.00 c/d), with 50 trials in each frequency. All trials from the two methods were intermixed. All observers participated in two experimental sessions, separated by 0.5 to 8 days. The results of the measurements were quantified in terms of the area under log contrast sensitivity function (AULCSF) and contrast sensitivities at the six spatial frequencies tested in the Ψ method. The results from the Ψ method were treated as the “truth” and used to evaluate the accuracy of the quick CSF method.

### Quick CSF Analysis

To obtain CSF estimates from each run of the quick CSF method, we sampled 1,000 sets of CSF parameters from the posterior distribution of CSF parameters, constructed 1,000 corresponding CSF curves, and obtained the empirical distribution of the CSF. This re-sampling procedure automatically takes into account the covariance structure in the posterior distribution of the CSF parameters, and allows us to assess the precision of the estimated CSF curve^50^.

## Results

### Experiment 1. Evaluation of quick CSF at an intermediate viewing distance (1.86 meters)

**Figure 1:**
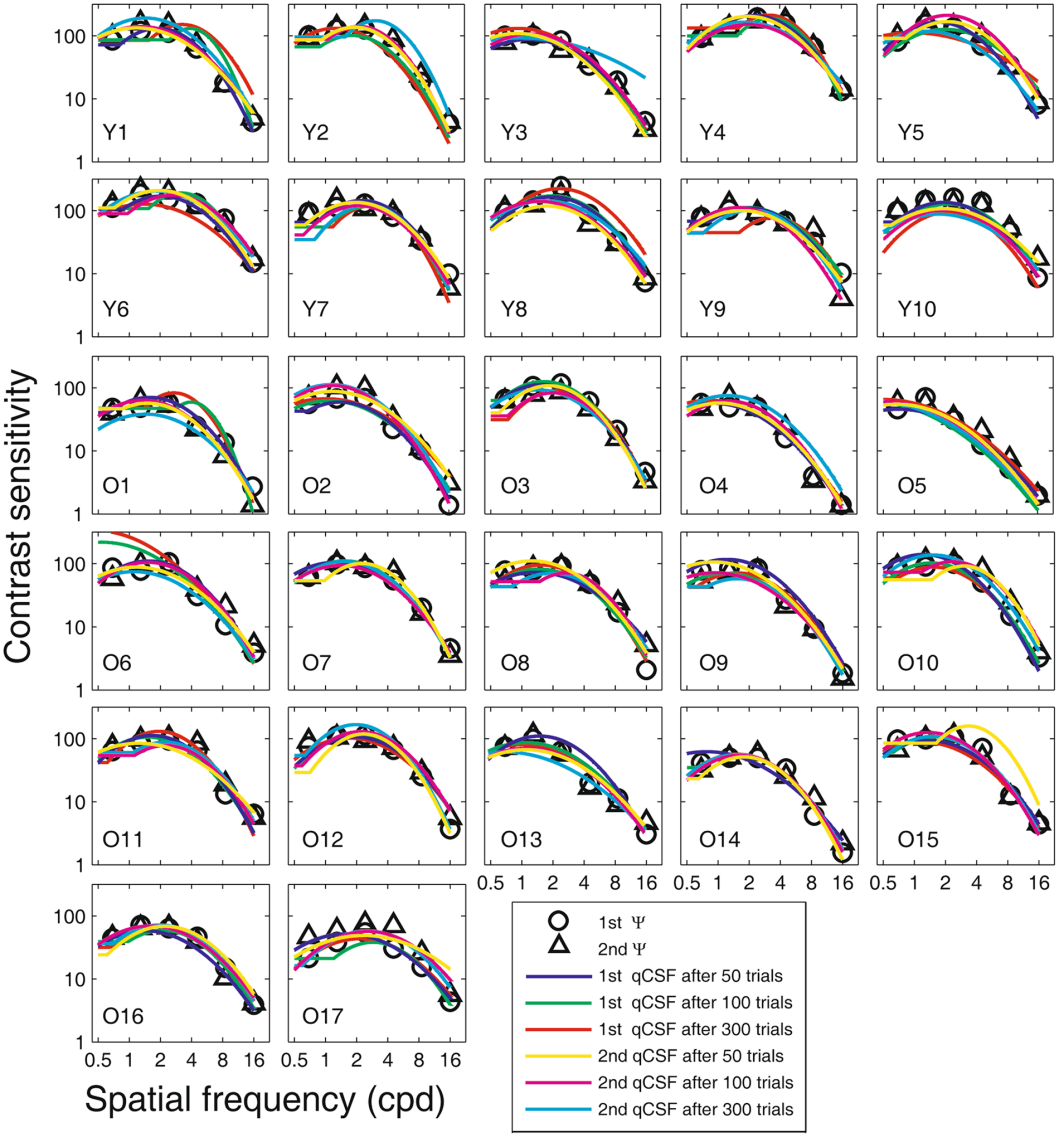
CSFs obtained from the quick CSF method and the Ψ method for each observer in two separate tests. The blue, green, and red curves represent CSFs estimated from 50, 100, and 300 trials of the quick CSF method in the first session of the experiment, respectively; the yellow, pink, and cyan curves represent CSFs estimated from 50, 100, and 300 trials of the quick CSF method in the second session of the experiment, respectively. Open circles and open triangles represent contrast sensitivities derived from the Ψ method in the first and second sessions. ‘Y’ denote the young group; ‘O’ denote the old group.

Figure 1 shows plots of CSFs estimated with 50, 100, 300 quick CSF trials and contrast sensitivities at the six spatial frequencies derived from the Ψ method in the first and second measurements. At the first glance, these CSFs overlapped with each other, indicating good reliability and consistency between the two methods. In the following sections, we evaluate the test-retest reliability of both CSF testing methods, examine the consistency of CSF estimates between the two methods, and check the assumption of the quick CSF implementation. We also assess the ability of the quick CSF method in distinguishing CSFs of the old from those of the young observers.

#### Precision

The precision of CSF estimates obtained with the quick CSF and Ψ methods was quantified by the average standard deviation of the distribution of resampled sensitivity estimates across all spatial frequencies for each observer:

2 $$Precision=\frac{\sum _{i=1}^{N}SDc{s}_{i}}{N},\,SDcs=\sqrt{\frac{\sum _{j=1}^{1000}{({{\rm{l}}{\rm{o}}{\rm{g}}}_{10}(C{S}_{j})-{{\rm{l}}{\rm{o}}{\rm{g}}}_{10}(\overline{CS}))}^{2}}{1000},}$$

where SD*cs* is the standard deviation of the distribution of resampled contrast sensitivity at each spatial frequency, *N* is the number of resampled spatial frequencies (N = 12), i indexes spatial frequency, and j indexes repetition. The precision index was calculated trial-by-trial and hence we will have 300 precision estimations for the quick CSF and the Ψ methods, respectively.

**Figure 2:**
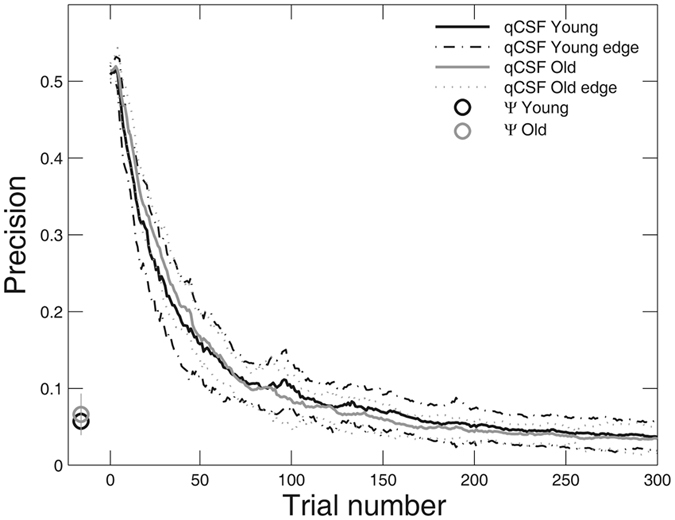
The average precision of the quick CSF method as a function of the number of trials (Young: black; Old: grey). The black and grey dotted curve lines indicate one standard deviation for the young and old groups, respectively. The average precisions of the Ψ method after 300 trials were marked in black (Young) and grey (Old) open circles; the error bar represents 1 SD.

For the quick CSF method, the precision was comparable in 93.7% and 100% of the 300 trials (p > 0.05, paired t-test) in the young and old group, respectively. Precision in the two groups didn’t differ significantly across most of the trials (89%, p > 0.05, independent-samples t-test). Figure 2 shows the precision as a function of trial number for the two groups, averaged across observers and sessions. For the young group, the precision was 0.158 ± 0.027 (mean ± sd.), 0.101 ± 0.020, and 0.036 ± 0.011 log_10_ units after 50, 100, 300 trials, respectively; for the old group, the precision was 0.168 ± 0.044, 0.085 ± 0.023, and 0.034 ± 0.017 log_10_ units after 50, 100, 300 trials. The precision of the Ψ method was 0.057 ± 0.011 log_10_ units for the young group and 0.066 ± 0.027 log_10_ units for the old group after 300 trials.

#### Test-Retest Reliability

**Figure 3:**
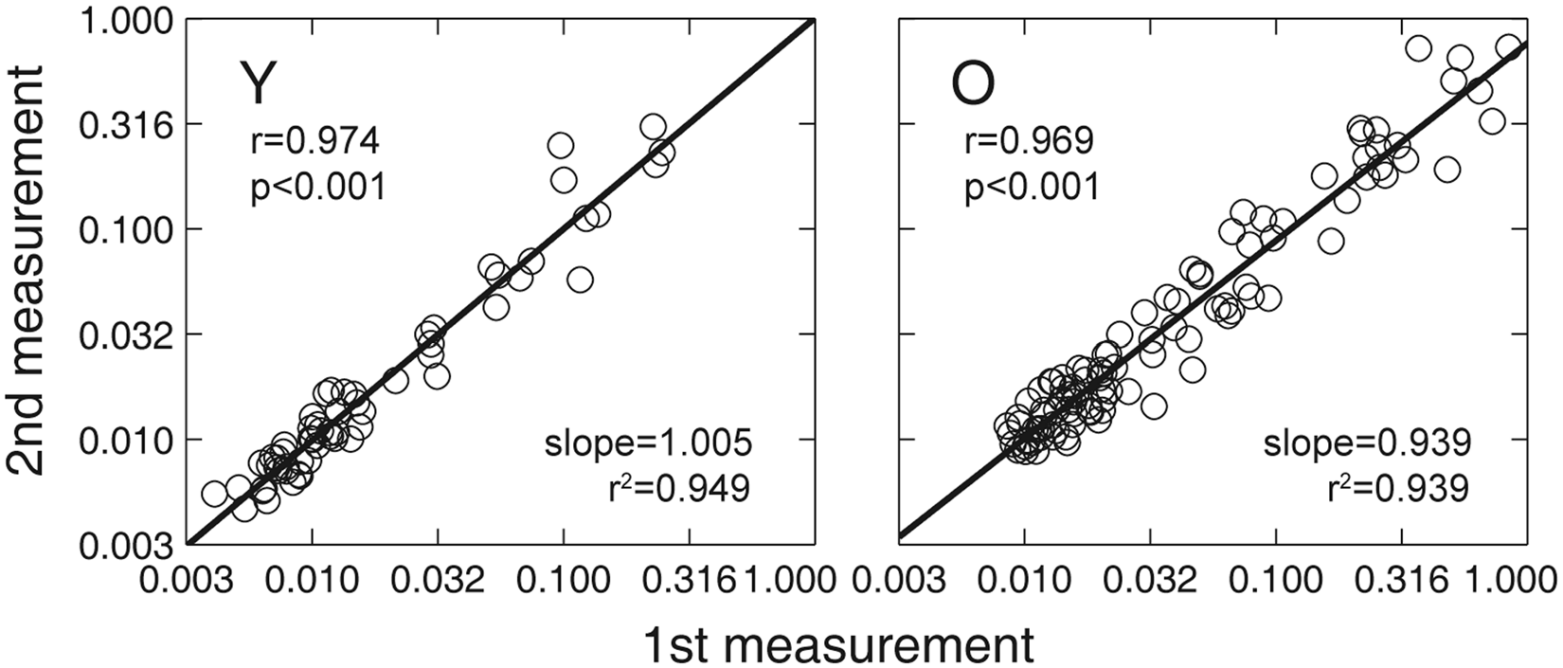
Comparison of estimated contrast thresholds at six spatial frequencies from the first and second Ψ tests of the young and old group. Data from all observers were pooled. ‘Y’ denote the young group; ‘O’ denote the old group.

We used Pearson correlation and linear regression analysis to evaluate the test-retest reliability of the Ψ method and the quick CSF method for the young and old observers. In Fig. 3, we plot contrast thresholds at six spatial frequencies in the first and second measurements of the Ψ method. The test-retest correlations for the Ψ method were 0.974 and 0.969 (both p < 0.001) for the young and old observers, respectively. Linear regression revealed a slope of 1.005 (confidence interval: [0.9439, 1.066], *r* ^2^ = 0.949) and 0.939 (CI: [0.892, 0.987], *r* ^2^ = 0.939) for the young and old observers, respectively.

**Figure 4:**
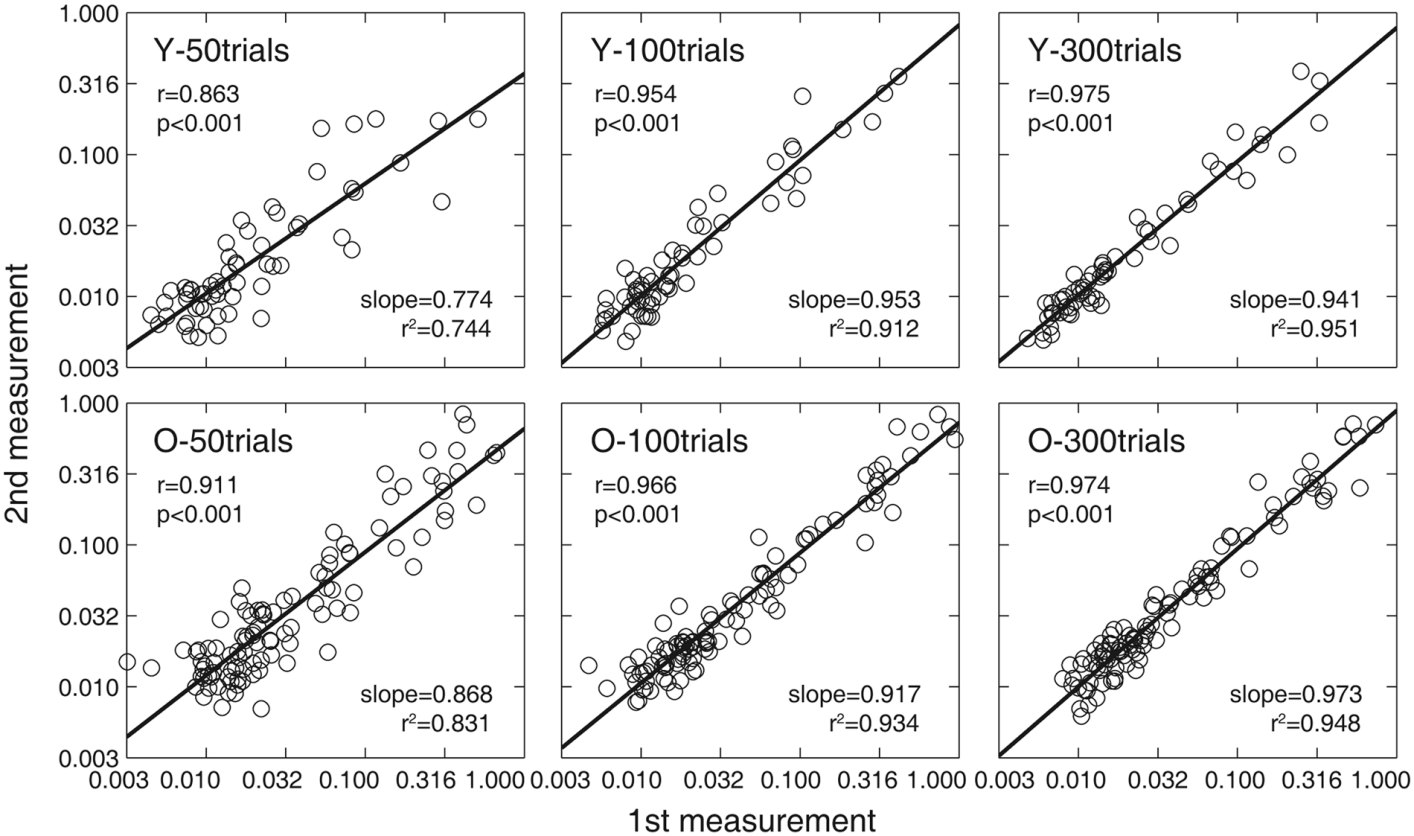
Contrast thresholds at six spatial frequencies after 50 (1^st^ column), 100 (2^nd^), and 300 (3^rd^) quick CSF trials in the first (x-axis) and second (y-axis) tests. Data from all observers were pooled. ‘Y’ denote the young group; ‘O’ denote the old group.

Contrast thresholds at six spatial frequencies after 50, 100, and 300 quick CSF trials for the young and old observers in the first experimental session are plotted against those in the second session in Fig. 4. The test-retest correlations for the young observers were 0.863 (p < 0.001), 0.954 (p < 0.001) and 0.975 (p < 0.001) after 50, 100, and 300 quick CSF trials, respectively. The results of linear regression showed that the slopes were 0.774 (CI: [0.655 0.894], *r* ^2^ = 0.744), 0.953 (CI: [0.875, 1.032], *r* ^2^ = 0.911), and 0.941 (CI: [0.885, 0.997], *r* ^2^ = 0.951) after 50, 100, and 300 quick CSF trials, respectively. For old observers, the test-retest correlations were 0.911, 0.966 and 0.974 (all p < 0.001) after 50, 100, and 300 trials, respectively. The slopes of linear regression were 0.868 (CI: [0.791 0.946], *r* ^2^ = 0.831), 0.917 (CI: [0.869 0.966], *r* ^2^ = 0.934), and 0.973 (CI: [0.928 1.018], *r* ^2^ = 0.948) after 50, 100 and 300 trials.

**Figure 5:**
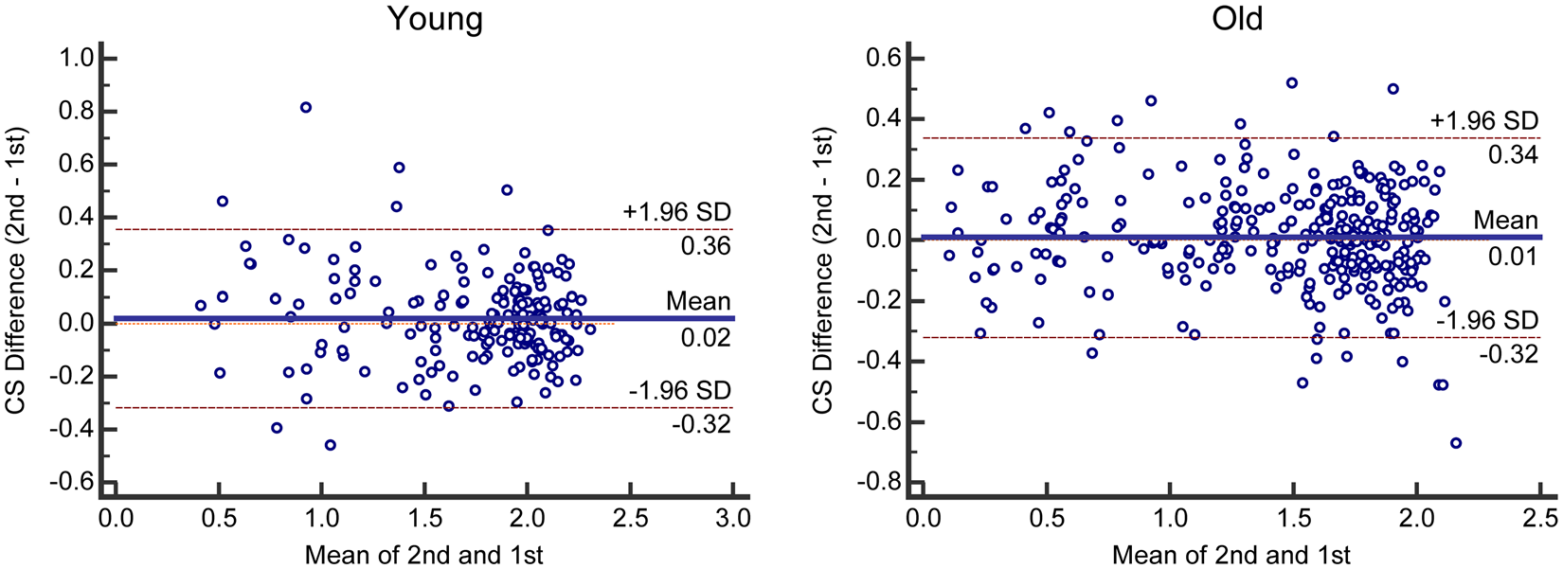
Bland-Altman plots of the test-retest reliability of the contrast sensitivity functions obtained with the quick CSF method for the young and old groups. The solid line represents the mean value of the difference between two tests and the dotted lines on the top and bottom represent 95% consistency boundaries. Circles represented the difference of contrast sensitivity at six spatial frequencies plotted against the means of the two measurements after 50,100,300 trials, respectively. CS: contrast sensitivity.

We also compared the test-retest reliability of the contrast sensitivity functions obtained with the quick CSF method using Bland-Altman plots^51^. Figure 5 shows that the difference of contrast sensitivity estimates between the first and second tests of the quick CSF procedure for the young and old groups fell within the 95% confidence limits. 3.89% and 5.88% of the scores were out of the 95% consistency boundaries in the young and old group, respectively. The Bland-Altman plot demonstrated high reliability of the quick CSF test in both group.

#### Quick CSF-Ψ Consistency

**Figure 6:**
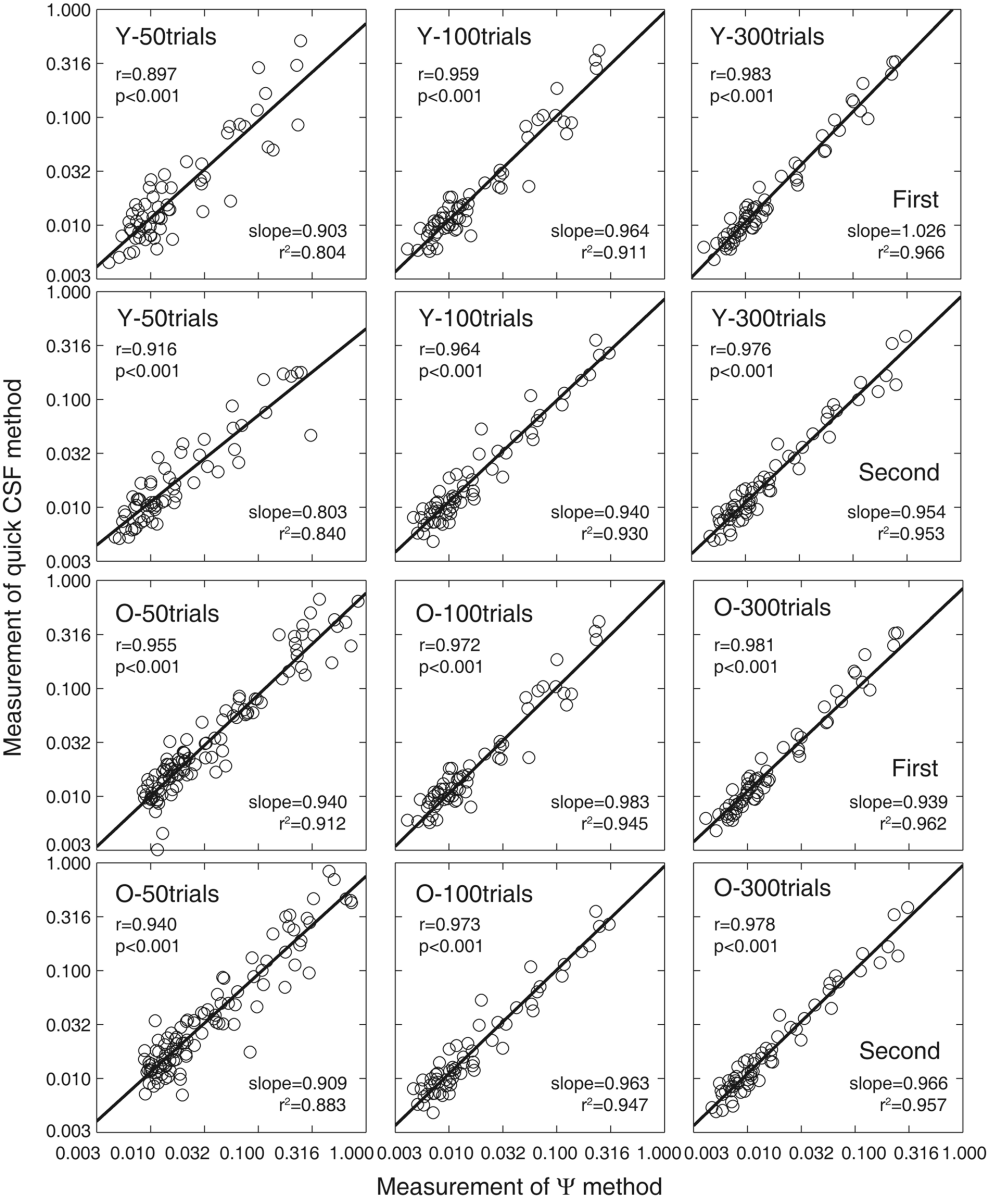
Contrast thresholds in six spatial frequencies after 50 (1^st^ column), 100 (2^nd^), and 300 (3^rd^) trials in the Ψ (x-axis) and quick CSF (y-axis) tests in the first (up) and second (down) sessions. Data from all observers were pooled. ‘Y’ denote the young group; ‘O’ denote the old group.

To evaluate the consistency between estimated CSFs from the quick CSF and Ψ methods, we conducted Pearson correlation and linear regression analysis on the contrast threshold for the young and old observers at six spatial frequencies in the first and second sessions (see Fig. 6). The absolute difference of contrast sensitivity between the two methods at each spatial frequency was calculated by

3 $${K}_{individual}=20|{\mathrm{log}}_{10}\frac{C{S}_{quickCSF}}{C{S}_{{\rm{\Psi }}}}|.$$

The mean absolute difference over spatial frequencies and observers in each group was computed as

4 $${K}_{group}=\frac{\sum {K}_{individual}}{M\ast N},$$

Where *M* is the number of observers and *N* is the number of spatial frequencies.

For young observers, the correlation coefficients of CSFs obtained from the quick CSF and Ψ methods were 0.897, 0.959 and 0.983 (all p < 0.001) after 50, 100, and 300 quick CSF trials in the first session and 0.916, 0.964 and 0.976 (all p < 0.001) in the second session. The results of linear regression showed that the slopes after 50, 100, and 300 quick CSF trials were 0.903 (CI: [0.786 1.02], *r* ^2^ = 0.804), 0.964 (CI: [0.889, 1.039], *r* ^2^ = 0.919), and 1.026 (CI: [0.975, 1.076], *r* ^2^ = 0.966) in the first session and 0.803 (CI: [0.711 0.895], r^2^ = 0.840), 0.940 (CI: [0.871, 1.007], r^2^ = 0.930), and 0.954 (CI: [0.898, 1.009], *r* ^2^ = 0.953) in the second session, respectively. The mean absolute difference of contrast sensitivity between the quick CSF and Ψ methods after 50, 100, and 300 trials were 3.336 ± 0.353 (mean ± se), 2.815 ± 0.340, and 2.408 ± 0.261 dB in the first session and 2.449 ± 0.333, 1.919 ± 0.217 and 1.528 ± 0.144 dB in the second session.

The correlation coefficients of the CSFs obtained from the two methods for the old observers in the first and second sessions were 0.955 and 0.940, 0.972 and 0.973, and 0.981 and 0.978 (all p < 0.001) after 50, 100, and 300 quick CSF trials, respectively. The results of linear regression in the first and second sessions showed that the slopes were 0.940 (CI: [0.883 0.9981], *r* ^2^ = 0.912) and 0.909 (CI: [0.843, 0.975], *r* ^2^ = 0.883), 0.983 (CI: [0.935, 1.03], *r* ^2^ = 0.945) and 0.963 (CI: [0.917, 1.008], *r* ^2^ = 0.947), and 0.939 (CI: [0.902 0.976], *r* ^2^ = 0.962) and 0.966 (CI: [0.926, 1.007], *r* ^2^ = 0.957) after 50, 100, and 300 quick CSF trials. The mean absolute difference of contrast sensitivity obtained from the two methods in the first and second sessions were 2.472 ± 0.218 and 2.768 ± 0.235, 1.791 ± 0.165 and 2.226 ± 0.199 dB, and 1.440 ± 0.142 and 1.332 ± 0.128 dB after 50, 100, and 300 quick CSF trials, respectively.

#### CSF Differences between the Young and Old Groups

**Figure 7:**
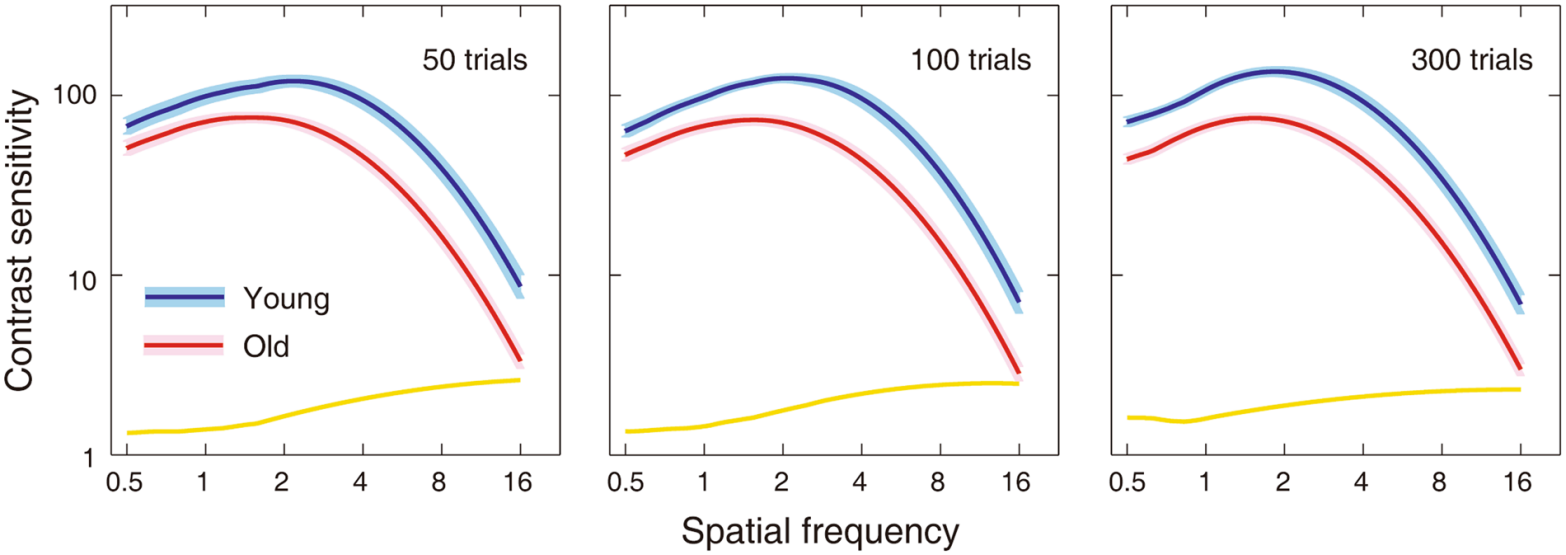
The average CSFs measured by the quick CSF method for the young (upper curve) and old (lower curve) groups after 50, 100, and 300 trials. The colored band indicates plus and minus one standard Error. The yellow curves represent the difference of log_10_ contrast sensitivity between the young and old group.

A between-observer analysis of variance (ANOVA) showed that there were highly significant differences in contrast sensitivity between the young and old observers after 50, 100, and 300 quick CSF trials (F(1, 25) = 26.794, 26.230, 29.716; all p < 0.001). A Post-hoc between-group t-test revealed that contrast sensitivity in the young group was significantly greater than that in the aged group for all tested frequencies (ranged from 0.5 to 16 c/d, t(25) = 1,858~5.426, 2.482~5.729, and 4.063~4.750 for 50, 100, and 300 trials, respectively; all p < 0.05). Furthermore, the interaction of spatial frequency and age was significant (F(1, 25) = 6.346, 4.902, and 2.863; all P < 0.05), indicating that the difference in contrast sensitivity varied with spatial frequency. Averaged over subjects, the difference in contrast sensitivity between the aged and young groups increased from 0.13 ± 0.02 for 50 trials, 0.13 ± 0.01 for 100 trials, 0.21 ± 0.01 for 300 trials at 0.5 c/d to 0.43 ± 0.02, 0.41 ± 0.02, and 0.37 ± 0.02 at 16 c/d, indicating greater degradation at higher frequencies. Figure 7 shows the estimated CSFs and their difference averaged across observers and testing sessions for the young (upper curve) and old (lower curve) groups. The results suggested aging may affect contrast sensitivity over a wide range of spatial frequencies, with greater impact at high spatial frequencies, consistent with Nameda, Kawara and Ohzu^52^, and Sloane, Owsley, & Alvarez^38^.

#### Classification between Old and Young Groups

The significant difference in CSF between the old and young groups prompted us to evaluate whether CSF is a good indicator of aging. We selected the Area Under the Log CSF (AULCSF), a summary metric of the CSF function^29, 33, 53, 54^, and cutoff spatial frequency (cutSF) that characterizes the high spatial frequency limit of spatial vision as predictors. The AULCSF was calculated as the integration of the area under the log CSF curve and above 0 from 0.5 to 16 c/d. The cutSF was defined as the spatial frequency at which contrast sensitivity is 1.0 (threshold = 1.0). We used a logistic regression analysis to predict membership in the two groups:

5 $$P(old\,|\,AULCSF,cutSF)=\frac{\exp ({\beta }_{0}+{\beta }_{1}\cdot AULCSF+{\beta }_{2}\cdot cutSF)}{1+\exp ({\beta }_{0}+{\beta }_{1}\cdot AULCSF+{\beta }_{2}\cdot cutSF)},$$

where *P*(old) is the probability of being an old observer, and *β*_0_, *β*_1_, *β*_2_ are the coefficients.

The accuracy of the prediction was 74.1%, 85.2% and 92.6% after 50, 100, and 300 quick CSF trials in the first session and 88.9%, 85.2%, and 85.2% in the second session. *β*_0_ and *β*_1_ (all p < 0.05) had significant impact on the prediction, but *β*_2_ (all p > 0.1) didn’t in almost all the conditions (in the first session, *β*_0_ and *β*_1_ were 25.486 and −2.454 after 50 quick CSF trials, 28.5 and −3.177 after 100 quick CSF trials, and 40.720 and −4.821 after 300 quick CSF trials; in the second session, *β*_0_ and *β*_1_ were 36.303 and −3.284 after 50 quick CSF trials, 35.978 and −3.894 after 100 quick CSF trials, and 42.160 and −2.970 after 300 quick CSF trials). That is, AULCSF was the key factor for discriminating CSFs from the two groups. The results of Hosmer and Lemeshow goodness-of-fit (H&L GOF) test showed that there were no significant difference between the predicted and the observed values (*X* ^2^_7_ = 6.111, 4.061, and 8.549 after 50, 100, and 300 quick CSF trials in the first session and 8.176, 2.545, and 8.610 in the second session, all p > 0.1).

#### Evaluating the Truncated Log Parabola Function Assumption

One assumption of the quick CSF method is that the CSF can be characterized by a truncated log-parabolic function. If the shape of the CSF in the aging population were not well described by the truncated log-parabolic function, the quick CSF method would not provide an accurate measure of the CSF in the aging population. To validate the assumption, we fit the contrast sensitivities at six spatial frequencies (obtained with theΨmethod) with the truncated log-parabolic function:

$$\mathrm{log}\,10[\mathrm{CSF}(\mathrm{sf})]=\{\begin{array}{c}\,log10({{\rm{\gamma }}}_{{\rm{\max }}})-\delta ,\,\,lo{g}_{10}(sf) < lo{g}_{10}({{\rm{f}}}_{{\rm{\max }}})-0.5\beta \sqrt{-\delta /log10(0.5)};\\ \begin{array}{c},\,\\ log10({{\rm{\gamma }}}_{{\rm{\max }}})+log10(0.5){[\frac{lo{g}_{10}(sf)-lo{g}_{10}({{\rm{f}}}_{{\rm{\max }}})}{0.5\beta }]}^{2},\,otherwise.\end{array}\end{array}$$

The mean *r* ^2^ in the young group was 0.95 ± 0.01 and 0.94 ± 0.01 after 300 trials in the two test sessions, respectively. Consistent with previous research^33, 34^, the truncated log-parabolic function provided an excellent description of the CSFs of the young population. For the old group, the mean r^2^ was 0.96 ± 0.01 and 0.94 ± 0.01 after 300 trials in the two test sessions, respectively. The truncated log-parabolic model provided an excellent description of the CSFs of the old population.

#### Evaluating the Constant Slope Assumption

In our implementation of the quick CSF method, there was one important assumption, that is, the slope of the psychometric function is invariant across spatial frequencies. A violation of this assumption might lead to evident bias and inaccuracy of evaluation. Validating the assumption is necessary for applications of the quick CSF method in the old population.

We binned the 100 Ψ trials for each spatial frequency from the two sessions into five equal contrast ranges (in log unit), calculated the fraction correct response corresponding to the mean of that range, and fitted a log-Weibull psychometric function (Equation 1) to the data in each spatial frequency condition. A maximum-likelihood procedure was used:

6 $$Likelihood={{\prod }_{i}\frac{{N}_{i}!}{{K}_{i}!({N}_{i}-{K}_{i})!}{P}_{i}^{{K}_{i}}(1-{P}_{i})}^{{N}_{i}-{K}_{i}},$$

where *i* denotes a contrast condition, *N*_*i*_ and *K*_*i*_ are the number of total and correct trials, and *P*_*i*_ is the fraction correct predicted by the log-Weibull function. The goodness-of-fit was then evaluated by *r* ^*2*^:

7 $${r}^{2}=1.0-\frac{\sum {({Y}_{predicted}-{Y}_{measured})}^{2}}{\sum {[{Y}_{measured}-mean({Y}_{measured})]}^{2}},$$

*where Y*_*predicted*_ is the model-predicted psychometric function, and *Y*_*measured*_ is the measured psychometric function.

A full model with six independent slopes at six different spatial frequencies and a reduced model with a single slope across six different spatial frequencies were compared with the *χ* ^2^ statistic:

8 $${\chi }^{2}(df)=2\,\mathrm{log}(\frac{likelihoo{d}_{full}}{likelihoo{d}_{reduced}}),$$

where *df* = *k*_full_ − *k*_reduced_. The *k*s are the number of parameters in each model.

**Figure 8:**
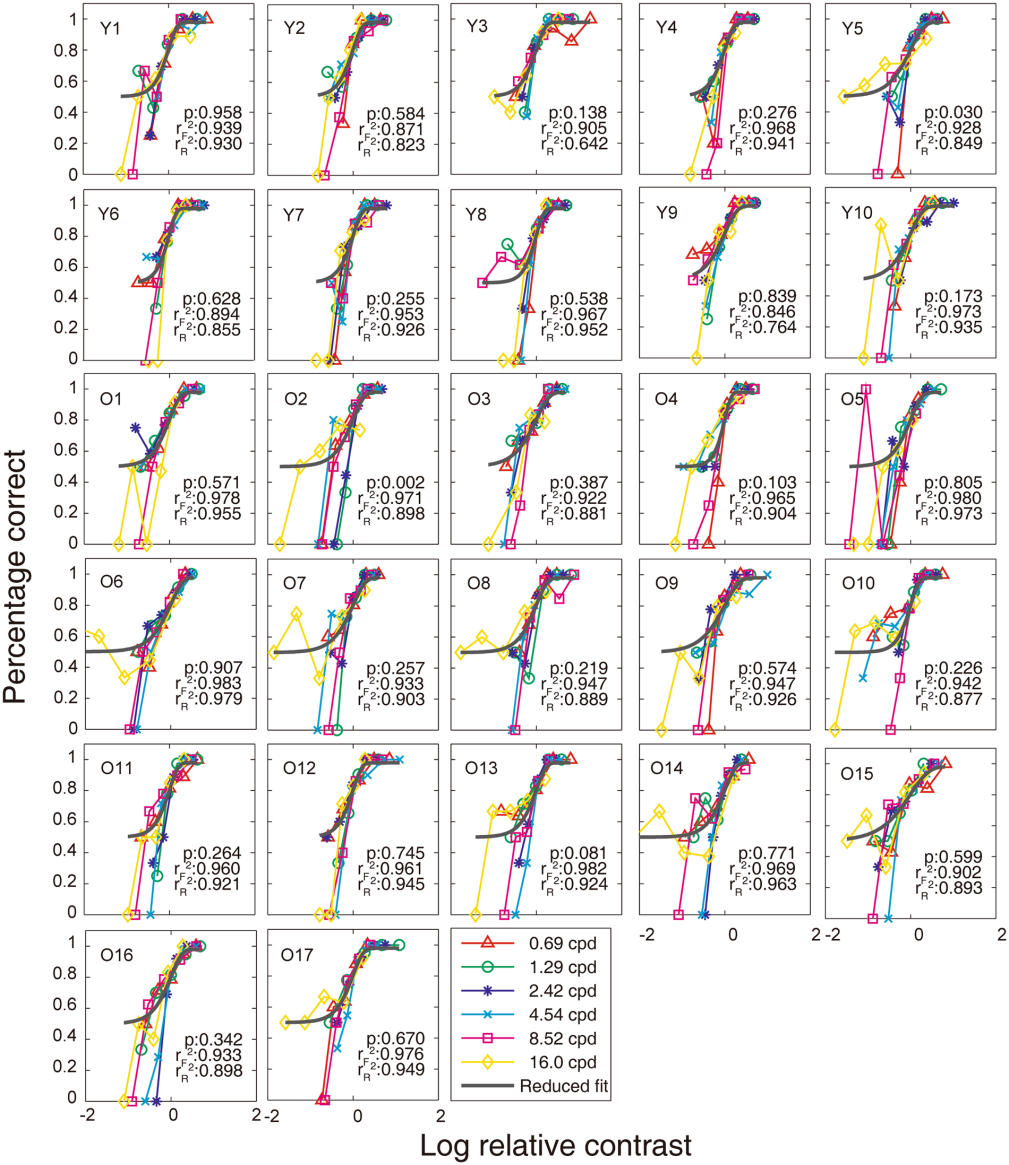
Raw psychometric functions in the six spatial frequency conditions obtained with the Ψ method. Data from each spatial frequency were normalized by that frequency’s threshold, and the predictions of the best-fitting single slope psychometric function are shown as curves. Only Y5 and O2 failed with slope invariance assumption. ${r}_{{\rm{F}}}^{2}$: the goodness-of-fit of the full model; ${r}_{{\rm{R}}}^{2}$: the goodness-of-fit of the reduced model.

Figure 8 depicts each observer’s psychometric functions in the six spatial frequency conditions and the fitted psychometric functions by the reduced model. Model comparison revealed that only observers Y5 and O2 failed the slope invariance test (p < 0.05). For all the other observers, the slope invariance assumption could not be rejected. Averaged over observers, the best-fitting slope of the reduced model was 1.94 ± 0.38 in the young group and 1.74 ± 0.41 in the old group, with no significant difference (t (25) = 1.276, p > 0.10). The average slope over the two groups was 1.81 ± 0.40.

### Experiment 2. Evaluation of quick CSF at a far viewing distance (4.29 meters)

**Figure 9:**
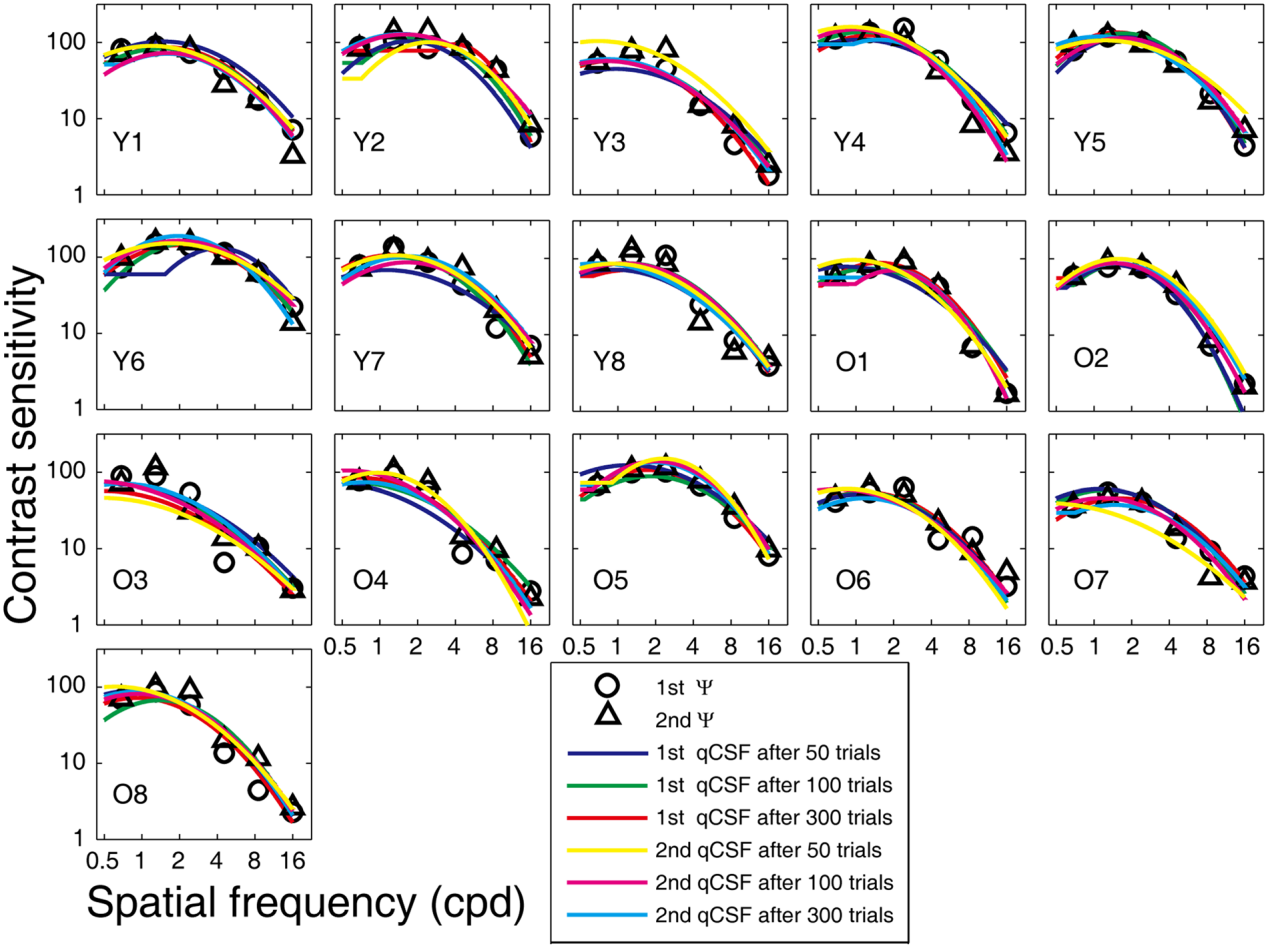
CSFs obtained from the quick CSF method and the Ψ method at 4.29 meters for each observer in two separate tests. The blue, green, and red curves represent CSFs estimated from 50, 100, and 300 quick CSF trials in the first session of the experiment, respectively; the yellow, pink, and cyan curves represent CSFs estimated from 50, 100, and 300 quick CSF trials in the second session of the experiment, respectively. Open circles and open triangles represent contrast sensitivities derived from the Ψ method in the first and second sessions. ‘Y’ denote the young group; ‘O’ denote the old group.

CSFs estimated with 50, 100, 300 quick CSF trials and contrast sensitivities at the six spatial frequencies derived from the Ψ method in the first and second measurements at the far viewing distance are shown in Fig. 9. Again, these CSFs overlapped with each other, indicating good reliability and consistency between the two methods.

For the young group, the precision was 0.158 ± 0.022 (mean ± sd.), 0.083 ± 0.014, and 0.026 ± 0.013 log_10_ units after 50, 100, 300 trials, respectively. The test-retest correlations for the young observers were 0.936 (p < 0.001), 0.972 (p < 0.001) and 0.975 (p < 0.001) after 50, 100, and 300 quick CSF trials, respectively. The slopes of linear regression were 0.942 (CI: [0.837 1.047], r^2^ = 0.876), 0.974(CI: [0.905, 1.044], r^2^ = 0.945), and 0.941 (CI: [0.877, 1.004], r^2^ = 0.951) after 50, 100, and 300 quick CSF trials, respectively. For the old group, the precision was 0.160 ± 0.035, 0.094 ± 0.029, and 0.038 ± 0.014 log_10_ units after 50, 100, 300 trials; the test-retest correlations were 0.949, 0.978 and 0.986 (all p < 0.001) after 50, 100, and 300 trials, respectively; the slopes of linear regression were 1.014 (CI: [0.907 1.121], *r* ^2^ = 0.888), 1.037 (CI: [0.964 1.111], *r* ^2^ = 0.946), and 1.022 (CI: [0.970 1.073], *r* ^2^ = 0.972) after 50, 100 and 300 trials. The correlation coefficients of CSFs obtained from the quick CSF and Ψ methods were 0.956, 0.977 and 0.983 (all p < 0.001) after 50, 100, and 300 quick CSF trials in the first session and 0.953, 0.965 and 0.971 (all p < 0.001) in the second session for the young observer. The correlation coefficients of the CSFs obtained from the two methods for the old observers in the first and second sessions were 0.921 and 0.917, 0.957 and 0.937, and 0.942 and 0.970 (all p < 0.001) after 50, 100, and 300 quick CSF trials, respectively.

Model comparison revealed that the slope invariance assumption could not be rejected (all p > 0.1). Averaged over observers, the best-fitting slope of the reduced model is 1.81 ± 0.52 in the young group and 1.73 ± 0.37 in the old group, with no significant difference (t (14) = 0.327, p > 0.10). The average slope over the two groups is 1.77 ± 0.44.

The Bland-Altman analysis showed that only 4.86% and 5.56% of the scores were out of the 95% consistency boundaries in the young and old groups, respectively.

**Figure 10:**
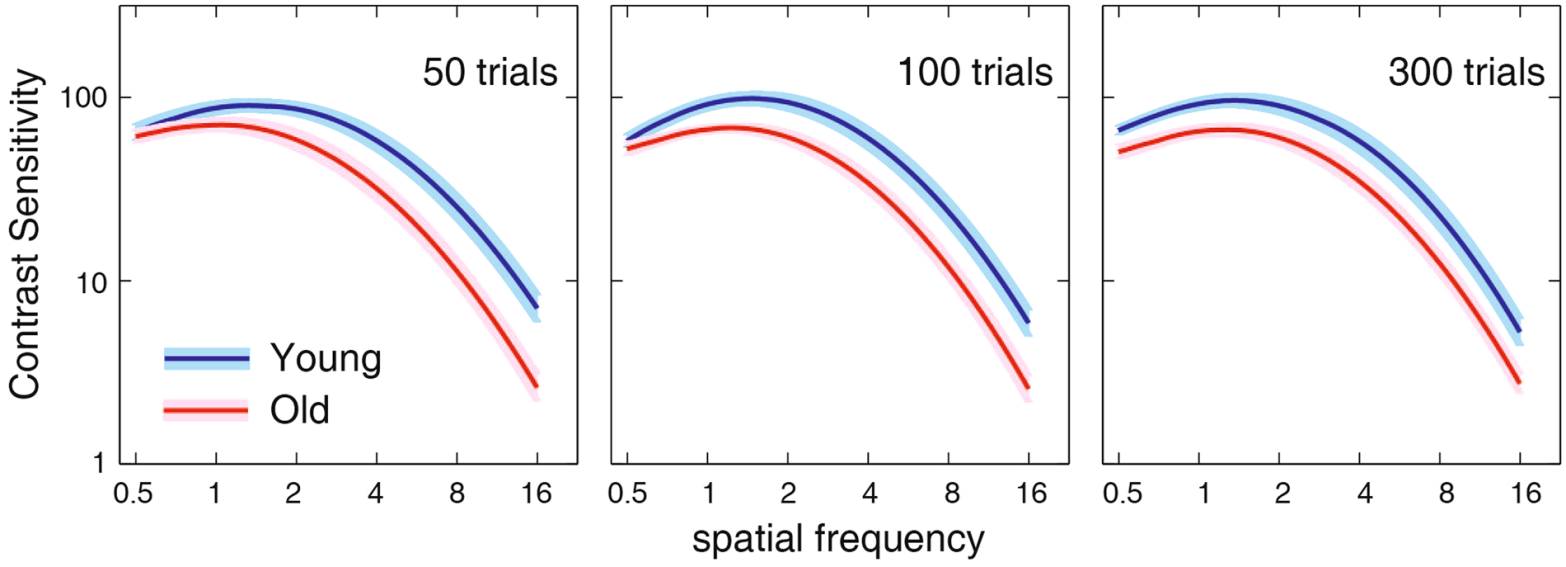
Average CSFs over observers and testing sessions measured by the quick CSF method for the young (upper curve) and old (lower curve) groups after 50, 100, and 300 trials (Experiment 2). The colored (gray) band indicates plus and minus one standard Error.

A between-observer analysis of variance (ANOVA) showed that there were highly significant differences in contrast sensitivity between the young and old observers after 50, 100, and 300 quick CSF trials (F(1, 14) = 4.554, 8.005, and 5.798; all P < 0.05). Figure 10 shows the estimated CSFs averaged across observers and testing sessions for the young (upper curve) and old (lower curve) groups at the far viewing distance. The results of logistic regression analysis showed that the accuracy of the group membership prediction was 81.3%, 87.5% and 81.3% after 50, 100, and 300 quick CSF trials. There was no significant difference in contrast sensitivity obtained with the intermediate and far viewing distances after 50, 100, and 300 quick CSF trials in the old groups (F(1, 23) = 1.860, 0.796, and 0.721; all *p*s > 0.10) and marginal difference in the young group (F(1, 16) = 3.434 (p = 0.082), 2.628 (p > 0.10), and 3.261(p = 0.090), respectively).

## Discussion

In this study, we evaluated the reliability, accuracy, and efficacy of the quick CSF method^29^ in the aging population. Our results showed that the quick CSF method can be used to derive a reasonable CSF estimate with as few as 50 trials in about 3 to 5 minutes. The CSFs obtained from the quick CSF method well matched those from the traditional Ψ method^43^, with exceptionally good test-retest reliability. Furthermore, the quick CSF method can reliably detect the CSF difference between the young and old groups.

There are two major assumptions in the implementation of the quick CSF procedure^29, 33^: (1) CSF can be described by a truncated log parabola function with four parameters, peak gain, peak spatial frequency, bandwidth, and truncation; and (2) the slope of the contrast psychometric function is invariant across spatial frequencies. Our results suggest that the CSF derived from the traditional Ψ methods can be well described by the truncated log parabola function. Also, we confirmed through model comparison that the slope invariance assumption was valid for almost all observers. Averaged over observers, the slope of contrast psychometric function was 1.94 ± 0.38 in the young group and 1.74 ± 0.41 in the old group at the intermediate viewing distance and 1.81 ± 0.52 in the young and 1.73 ± 0.37 in the old group at the far viewing distance, with no significant difference, consistent with findings in other studies^33, 55^. Taken together, these results paved the way for using the quick CSF procedure in the aging population.

Test-retest correlations appeared to be higher in the older group, that is 0.911 vs 0.863 after 50 trials, 0.966 vs 0.954 after 100 trials, and 0.974 vs 0.975 after 300 trials at the intermediate viewing distance and 0.949 vs 0.936, 0.978 vs 0.972 and 0.986 vs 0.975 at the far viewing distance, particularly with reduced numbers of trials (i.e. 0.911 vs 0.863). We didn’t exactly know the reason. Our observation was that the old observers were more serious participants and were generally more alert during the experiment, consistent with the personality studies that showed that older adults are generally more conscientious than their younger counterparts^56, 57^.

It’s important to note that the old observers might potentially experience presbyopic blur due to less vergence/accommodation ability at the intermediate viewing distance^44, 45^ in Experiment 1. To rule out this possibility, we conducted Experiment 2 at a far viewing distance, keeping all other experimental setups identical to those of Experiment 1. In general, all major conclusions remained the same when applying the quick CSF procedure at the far distance. Meanwhile, there was no significant difference in contrast sensitivity obtained at the intermediate and far viewing distances after 50, 100, and 300 quick CSF trials in both the young and old groups.

Although it’s commonly accepted that aging degrades contrast sensitivity, the spatial frequency selectivity of the degradation is still controversial. In the current study, we found that contrast sensitivity declined across all spatial frequencies in the aging population and the impact was more profound at high spatial frequencies. By producing interference fringe patterns directly on observers’ retinas, Nameda, Kawara, and Ohzu^52^ found that contrast sensitivity decreased at all frequencies for 50–60 year old observers, but it only decreased at high spatial frequencies for 40 year old observers. Owsley, Sekuler, and Siemsen^39^ showed that contrast sensitivity decreased only in high spatial frequencies (>2 c/d) for 40–50 year old observers. In a later study, Sloane, Owsley, and Alvarez^38^ reported that contrast sensitivity decreased at all spatial frequencies and the decline was particularly marked in high spatial frequencies. They attributed the different results to different stimuli (stationary vs flicker grating) or stimuli sizes (4° vs 6°) used in the studies. In addition, Crassini, Brown and Bowman^3^ claimed that young observers only had better contrast sensitivities in relatively high spatial frequencies (e.g. 2.0 and 5.0 c/d) but not in low frequencies (e.g. 0.2 and 0.8 c/d). It’s important to note that stimulus characteristics (e.g. static or dynamic, size), stimulus delivery method (e.g. interference fringe, monocular, and binocular), age variability (40~80 years), and screening criteria (ruling out ophthalmological diseases or not) are different among the different studies in the literature, which may have contributed to the apparent discrepancies in the observed contrast sensitivity deficits in old observers.

What caused the decline of contrast sensitivity in aging? There are three possible explanations. First, the degradation of contrast sensitivity may simply reflect decreased visual acuity. In Experiment 1 of the current study, we did find that logMAR acuity differed between the aged and young groups (t(25) = −5.550, *p* < 0.001). However, although there was significant correlation between AULCSF and visual acuity in the young group (*r* = −0.807 and −0.812 after 100 and 300 quick CSF trials, both *p* < 0.05; *r* = −0.545, *p* > 0.1 after 50 trials), AULCSF didn’t correlate with visual acuity in the old group (*r* = −0.205, −0.239, and −0.264 after 50, 100 and 300 quick CSF trials; *p* > 0.1), indicating that the degradation in contrast sensitivity in the old group cannot be solely accounted for by the decline in visual acuity. In Experiment 2, we found similar results (young group: *r* = −0.808 and 0.861 after 100 and 300 quick CSF trials, both *p* < 0.05; instead, old group: *r* = −0.338 and 0.299 after 100 and 300 quick CSF trials, both *p* > 0.10). These results, taken together with previous reports^58, 59^, indicate that the contrast sensitivity function provides additional information to that provided by visual acuity in the healthy aging population. Note that we set a logMAR normal or corrected visual acuity of better than 0.10 as one of the inclusion criteria (actually, only two old observers has log MAR acuity >0), which is more strict than that used in other study^60^.

Second, decline of contrast sensitivity may come from optics-related degradation^5, 39, 59, 61^, e.g., reduction of retinal illumination due to smaller pupil size^39, 62^, lower optical density of the ocular media^63^, and lens absorption^64^ in the aging population. The nature and extent of optical degradation on contrast sensitivity are still under debate. Generating interference fringe patterns using laser interferometry directly on the retina, Burton, Owsley, and Sloane^5^ found that the contrast sensitivity difference between the young and old adults shrunk greatly, implying that optical defects may be a major contributor to old observers’ sensitivity loss at photopic levels. With a similar method of stimulus presentation but a smaller visual field to bypass pupil size limitations and optical defects, Nameda, Kawara, and Ohzu^52^ got an opposite result that suggested there was no significant contribution of lens coloring on age-related decline of contrast sensitivity. Furthermore, the influence of senile miosis on the CSF of the elderly has been excluded by Sloane, Owsley, and Alvarez^38^. Bennett, Sekuler, and Ozin^41^ concluded that the decline of contrast sensitivity was not from reduced retinal illumination in aging.

Finally, aging may impact neural processing of contrast signals^4^. Using the linear amplification model (LAM), Pardhan, Gilchrist, Elliott, and Beh^65^ found that the decline of contrast sensitivity at a single spatial frequency of 6 c/d in old observers resulted from lowered neural calculation efficiency without any significant increase of internal additive noise. The result was supported by Bennett, Sekuler, and Ozin^41^ who conducted the test at 1, 3, and 9 c/d. They postulated that higher multiplicative noise and a mismatch between the spatiotemporal characteristics of the stimulus and the neural filter used to detect it might lead to lowered calculation efficiency. Pardhan^42^ further demonstrated that the decrease of visual function in old observers depended on the spatial frequency tested, with significantly lower calculation efficiencies at 1 and 4 c/d, and higher equivalent noise at 10 c/d. However, Allard, Renaud, Molinatti & Faubert^66^ found that healthy aging didn’t affect calculation efficiency in low spatial frequencies. They attributed the difference between the studies to the different spatial-temporal properties of the external noise patterns used in them. Electrophysiological studies from animals have attributed age-related losses in visual sensitivity to the decline of GABAergic inhibition in the aged visual cortex^67, 68^, which may lead to a non-specific increase of neuron firing rate and therefore a decrease in signal-to-noise ratio. In addition, the direct morphological evidence from Hua and his colleages^68^ showed that the density of GABA-immunorective neurons in the striate visual cortex of old cats is significantly lower than that of young adult cats, supporting the GABA hypothesis.

To conclude, we show the quick CSF method, which adopts a Bayesian information-gain strategy to greatly reduce CSF testing time (50~100 trials, 3~5 min), can provide an effective and reliable estimate of contrast sensitivity in the elderly. The method has demonstrated great potentials for both laboratory research and clinical evaluation in the aging population.
