# Author Correction: Efficient Characterization and Classification of Contrast Sensitivity Functions in Aging

**DOI:** 10.1038/s41598-019-42527-w

**Published:** 2019-04-12

**Authors:** Fang-Fang Yan, Fang Hou, Zhong-Lin Lu, Xiaopeng Hu, Chang-Bing Huang

**Affiliations:** 1 CAS Key Laboratory of Behavioral Science, Institute of Psychology, Chinese Academy of Sciences, Beijing, 100101 China; 2 Department of Psychology, University of Chinese Academy of Sciences, Beijing, 100101 China; 3 Laboratory of Brain Processes (LOBES), Center for Cognitive and Brain Sciences, Center for Behavioral and Cognitive Brain Imaging, and Department of Psychology, The Ohio State University, Ohio, 43210 USA; 4 Department of Radiology, The First Affiliated Hospital of Anhui Medicial University, HeFei, Anhui 230022 China

Correction to: *Scientific Reports* 10.1038/s41598-017-05294-0, published online 11 July 2017

This Article contains errors.

In the Methods section, under the subheading ‘Quick CSF Analysis’,

“This re-sampling procedure automatically takes into account the covariance structure in the posterior distribution of the CSF parameters, and allows us to assess the precision of the estimated CSF curve^50^.”

should read:

“This re-sampling procedure automatically takes into account the covariance structure in the posterior distribution of the CSF parameters, and allows us to assess the precision of the estimated CSF curve^30^.”

In addition, Reference 66 is incorrectly listed as ‘Arena, Hutchinson, Shimozaki & Long Visual discrimination in noise: behavioural correlates of age-related cortical decline. *Behav Brain Res* **243**, 102–8 (2013)’. The correct Reference 66 appears below as Reference^1^.
